# First Principle Study on Electronic and Transport Properties of Finite-Length Nanoribbons and Nanodiscs for Selected Two-Dimensional Materials

**DOI:** 10.3390/molecules27072228

**Published:** 2022-03-29

**Authors:** Mirali Jafari, Anna Dyrdał

**Affiliations:** Department of Mesoscopic Physics, ISQI, Faculty of Physics, Adam Mickiewicz University, ul. Uniwersytetu Poznańskiego 2, 61-614 Poznań, Poland; mirali.jafari@amu.edu.pl

**Keywords:** density functional theory, nanodiscs, finite-length nanoribbons, zero-energy states, 2D materials, zigzag trigonal nanodiscs

## Abstract

Using the density functional theory, we calculate electronic states of various nanoribbons and nanodiscs formed from selected two-dimensional materials, such as graphene, silicene, and hexagonal boron nitride. The main objective of the analysis is a search for zero-energy states in such systems, which is an important issue as their presence indicates certain topological properties associated with chirality. The analysis is also supported by calculating transport properties.

## 1. Introduction

The two-dimensional (2D) materials, such as graphene, TMDCs, and other van der Waals crystals, have turned out to be very promising materials for applications in nanoelectronics and photonics [1,2,3,4,5,6]. The unique electronic band structure of graphene leads to interesting quantum phenomena, such as the anomalous quantum Hall effect and series effects reserved for massless Dirac fermions [7,8]. Moreover, unique electronic properties, such as massless charge carriers, high carrier mobility, and controllable carrier density, make graphene the most promising platform for next-generation electronic devices [9].

**Figure 1:**
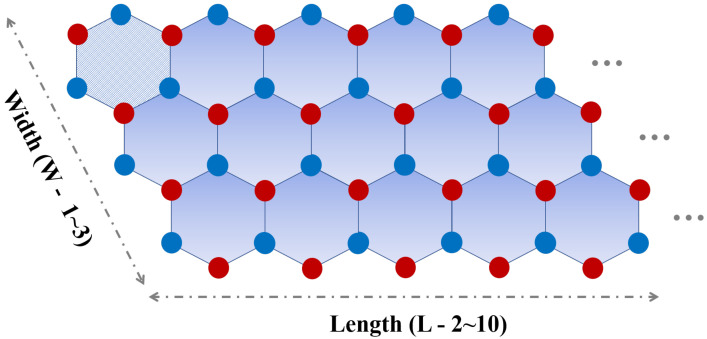
General schematic of the zigzag finite-length nanoribbons with different widths (W) and lengths (L). The nanoribbons with equal W and L may be regarded as parallelogrammic nanodiscs. The blue and red circles correspond to atoms in different sublattices, and to boron (B) and nitrogen (N) atoms for h-BN, respectively. A single hexagon is the basic element for the construction of zigzag nanoribbons.

**Figure 2:**
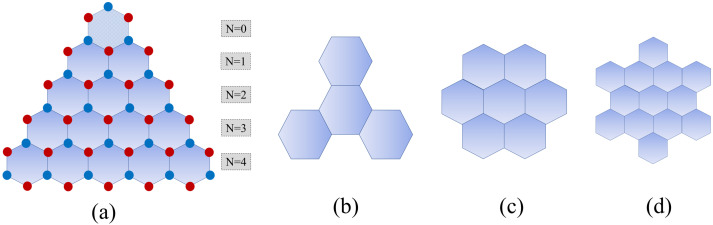
General schematics of the selected 0D nanodiscs; (**a**) zigzag trigonal nanodiscs of different widths from N = 0 to N = 4, (**b**) armchair trigonal nanodisc, (**c**) zigzag hexagonal nanodisc, and (**d**) armchair hexagonal nanodisc. The blue and red circles correspond to different sublattices, and to Boron (B) and Nitrogen (N) atoms for h-BN, respectively. An individual hexagon is the basic element of the nanodiscs’ construction.

Significant attention is also focused on graphene nanoribbons, in which a wide variety of band-gaps can be observed—from metallic to semiconducting ones [10,11,12,13,14]. Such nanoribbons can be fabricated using, for instance, patterning methods based on lithography [15,16,17]. Interestingly, the edge engineering affects the electronic and magnetic properties of nanoribbons. It has been found that when the edges of graphene are of the zigzag type, a half-filled flat band appears at the zero-energy (Fermi) level that is responsible for metallic and ferromagnetic properties [18,19,20]. Although a lot of theoretical research has been focused on infinite (long) nanoribbons, in reality they are of finite size. Their geometric configuration is shown schematically in Figure 1. Such finite-length (especially short) nanoribbons can be considered as zero-dimensional (0D) nanosystems. However, apart from the finite-length nanoribbons, there are also other 0D graphene derivatives with nano-sized disc-shaped close-edged forms, known as nanoflakes, nanoislands, or nanodiscs. Some of them have already been designed, fabricated, and then studied theoretically as well as experimentally [21,22,23,24,25]. Figure 2 illustrates several types of graphene nanodiscs that can be constructed by connecting a number of basic hexagons. Note that short nanoribbons can be considered as a specific sort of nanodisc.

Even though most of the research on two-dimensional structures has been carried out on graphene, it is not the only 2D material. Following graphene, many other two-dimensional graphene-like materials have been synthesized, such as, for instance, silicene, hexagonal boron nitride (h-BN), transition metal dichalcogenides (TMDs), oxides, and carbides [26,27,28,29,30,31,32,33,34,35,36,37,38]. It is also worth noting that most 2D materials occur in mono-layer as well as multi-layer forms [39,40,41,42,43,44,45].

This work focuses on nanodiscs and finite-length nanoribbons of graphene, silicene, and h-BN and particularly on their electronic properties studied by the density functional theory (DFT). It is worth noting that a comprehensive analytical tight-binding model of nanodiscs has been studied previously by Ezawa [46,47,48,49,50]. Since nanoribbons of h-BN (either with zigzag or armchair edges) are insulators, one may assume that all forms of these materials should be insulating as well, and our results confirm this hypothesis [51,52]. Similarly, because of the metallic behavior of all long graphene and silicene zigzag nanoribbons, one might expect similar behavior in other forms of these materials. However, there is scarce evidence of the zero-energy states in 0D forms of these materials. Our results demonstrate the absence of zero-energy states in finite-length nanoribbons with zigzag edges. In turn, for all studied graphene and silicene nanodiscs with trigonal and hexagonal geometries and zigzag/armchair edges, only those with trigonal geometry and zigzag edges exhibit half-filled zero-energy states and can be considered as metallic.

To support the above conclusions, we also consider transport properties of nanodisc–lead devices, where the electrodes are made of zigzag graphene, silicene, and h-BN ribbons, passivated with hydrogen atoms. The central region has been selected as a zigzag trigonal nanodisc as this is the only form of the studied nanodiscs that exhibits the zero-energy states. The remaining part of this article is as follows. Section 2 presents the methods and computational details. Section 3 describes the electronic properties of finite-length nanoribbons and nanodiscs. In turn, transport properties of zigzag trigonal nanodiscs are considered in Section 4. Finally, the conclusions are given in Section 5.

## 2. Model and Method

### 2.1. Model

In this paper, we restrict our considerations to three stable 2D materials: graphene, silicene, and h-BN. As already mentioned above, graphene is composed of carbon atoms with a honeycomb structure of hexagons. In the simulation codes, a zero-dimensional nanodisc can be made either from a finite-length nanoribbon or from individual hexagons. This also applies to silicene and h-BN. To maintain stability, the edges of all structures have been passivated with hydrogen atoms.

In turn, for the transport analysis, the device is made from three parts, including the right and left electrodes as well as the central scattering region. Electrodes in such a case are in the form of long (semi-infinite) nanoribbons of silicene, graphene, and h-BN. These two leads are connected to the central part formed by the zigzag trigonal nanodiscs of various sizes and shapes.

### 2.2. Computational Details

All calculations performed in this work have been carried out based on the density functional theory (DFT) [53] within the linear combination of atomic orbital (LCAO) method as implemented in the Quantum ATK code package (version 2021.06-SP2) [54,55,56]. To expand the wave function, the PseudoDojo collection of optimized norm-conserving Vanderbilt (ONCV) pseudopotentials with the ultra (LCAO-U) [54] basis set has been employed. The energy cutoff of 600 Rydberg has been assumed. For exchange–correlation potential calculations, generalized gradient approximation (GGA) with Perdew–Burke–Ernzerhof (PBE) [57] has been used. The considered geometries in this paper are molecules, hence a large simulation box of dimensions 25×25×25 Å has been used to prevent any interactions between periodic images. All of our calculations have been fully relaxed using the geometry optimization through the limited memory Broyden–Fletcher–Goldfarb–Shannon algorithm (LBFGS), with $10^{-3}$ eV/Å force tolerance and 0.1 GPa stress error tolerance. Additionally, relative convergence for the self-consistent field (SCF) energy was reached until $10^{-5}$ eV/Å (see also Section II in the Supplementary Materials). The Brillouin zone sampling for the electronic properties was Γ (i.e., 1×1×1) due to the lack of any periodic interlayer interactions. The Monkhorst–Pack grid [58] for transport calculations was also set to 1×1×380 after proper convergence.

Calculations of the transport properties are based on the DFT approach, coupled with the non-equilibrium Green’s function (NEGF) technique [59,60]. Based on the Landauer–Büttiker formalism, the current–voltage (I−V) characteristics of the system are determined, while the current flowing in the system is calculated from the following formula:

(1) $$I=\frac{2e}{h}\int T\left(E,V\right)\left[f_{L}\left(E\right)-f_{R}\left(E\right)\right]dE,$$

where T(E,V) is the transmission function of electrons of energy *E* through the central region from the left to the right electrodes, $f_{L}\left(E\right)$ and $f_{R}\left(E\right)$ are Fermi distribution functions of left and right leads, while *V* is the applied voltage.

## 3. Electronic Properties

### 3.1. Finite-Length Nanoribbons

**Figure 3:**
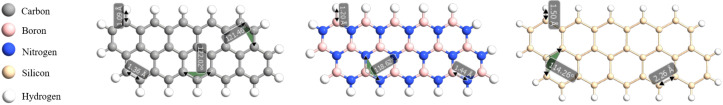
The optimized structure of the zigzag finite-length nanoribbons of (**left**) graphene, (**middle**) h-BN, and (**right**) silicene for (W, L) = (2, 5).

In this section, we investigate the electronic properties of finite-length zigzag nanoribbons of graphene, silicene, and h-BN, and especially the zero-energy states. Such zero-energy states are important because they remain zero energy as long as the chiral symmetry is preserved. For constructing finite-length nanoribbons, we make a primitive hexagon (benzene shaped), which is a unit cell of the considered 2D materials. Then, to generate a nanoribbon we repeat it along the width (W) and along the length (L). A finite-length nanoribbon can be considered as a parallelogrammic nanodisc, when its length and width are equal. For stability of the considered structures, the edges have been passivated with hydrogen atoms. To obtain a clear view, the optimized structures of the graphene, silicene, and h-BN finite-length nanoribbons are shown in Figure 3 for W = 2 and L = 5.

**Figure 4:**
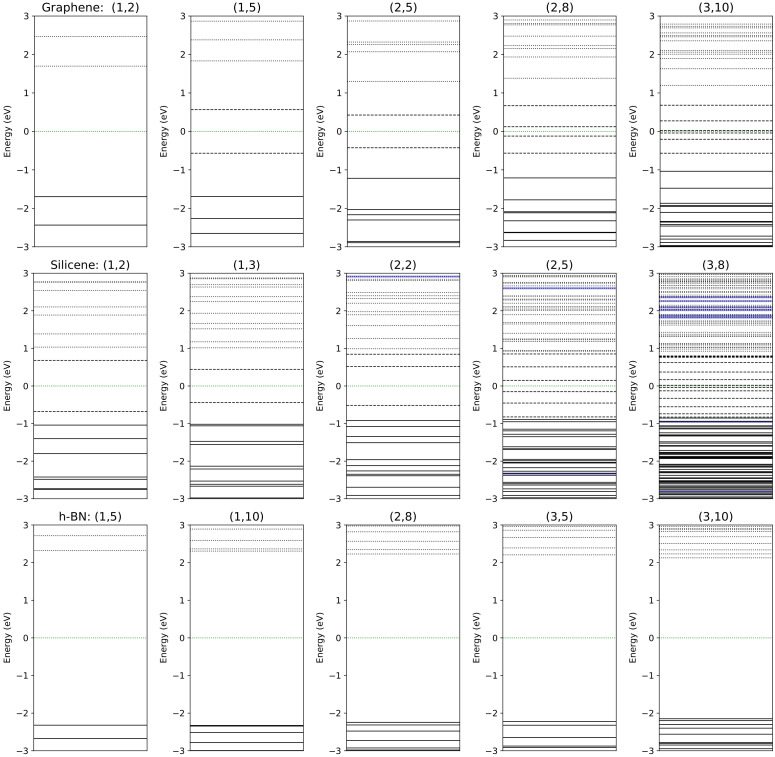
The full energy spectrum of the zigzag finite-length nanoribbons for (**top**) graphene, (**middle**) silicene, and (**bottom**) h-BN.

**Figure 5:**
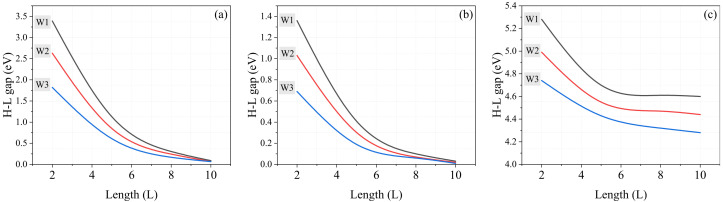
HOMO–LUMO band-gap behavior as a function of the length and width for (**a**) graphene, (**b**) silicene, and (**c**) h-BN finite-length zigzag nanoribbons.

We use the total density of states (TDOS) and the energy spectrum diagram to check the presence/absence of the zero-energy states. Some of these diagrams are displayed in Figure 4. The top panel of Figure 4 refers to the finite-length zigzag nanoribbon of graphene, from which the absence of zero-energy states is clearly evident. Our calculations are in good agreement with the tight-binding (TB) model Hamiltonian [46,47]. Another crucial point concerns behavior of the energy band-gap as a function of the nanoribbon’s length. From Figure 5, it is evident that the HOMO–LUMO energy gap—energy differences between the highest occupied molecular orbital and the lowest unoccupied molecular orbital—decreases with increasing length. Therefore, one can expect to have zero-energy states in sufficiently long nanoribbons. Moreover, with increasing width of the nanoribbons, the gap reduction with increasing *L* is faster. Hence, the metallic behavior of these structures depends on their length and width, which is consistent with the fact that infinite-length nanoribbons have a flat band of degenerate zero-energy states [18,19]. The full-energy spectra of silicene finite-length zigzag nanoribbons are shown in the middle panel of Figure 4. The main conclusion is that there are no zero-energy states and the gap decreases with increasing length and width of the nanoribbons (see Figure 5), similarly as in the case of graphene nanoribbons. However, silicene nanoribons have a smaller energy gap than similar graphene nanoribbons, so one may expect zero-energy states in sufficiently long nanoribbons.

We have also investigated the zigzag h-BN nanoribbons of finite length, and checked the behavior of the gap with the length *L* and width *W*, see Figure 5. This material is isomorphic and isoelectronic with the graphene honeycomb lattice. However, the difference is that h-BN exhibits a very wide band-gap, even for large lengths of nanoribbons, which can be concluded from Figure 4 (bottom panel). In contrast to the infinite nanoribbons of graphene, the h-BN nanoribbons display good semiconductor behavior [61,62].

### 3.2. Nanodiscs

**Figure 6:**
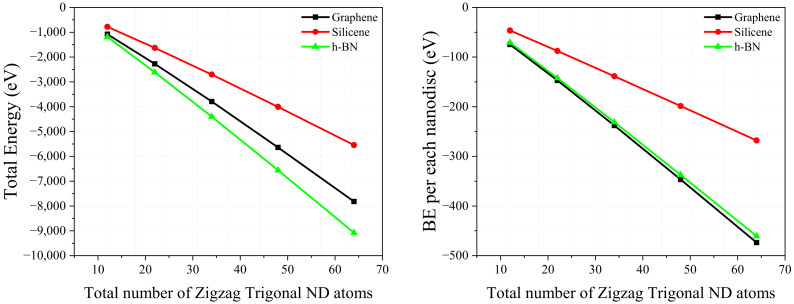
Behaviorof the total energy and binding energy as a function of the total number of constituent atoms of the zigzag trigonal nanodiscs.

To search further for zero-energy states, we study the electronic properties of the nanodiscs of various shapes and sizes, and for the selected 2D materials—graphene, silicene, and h-BN. Each nanodisc is constructed by connecting a certain number of hexagons. Due to a large variety of nanodiscs in terms of size and shape, we investigate only a few selected nanodiscs which are shown in Figure 2. The geometric configurations of the considered nanodiscs are dynamically stable. The corresponding binding energies are large and negative (see Table 1), which confirm stability of the structures. As expected, magnitude of the binding energy increases with the size of the nanodiscs. Figure 6 shows variation in the total energy with increasing size of the zigzag trigonal nanodiscs. As one can conclude, the structures after full relaxation are thermodynamically stable. We note that the binding energy of the nanodiscs can be calculated as:

(2) $$E_{b}\left(eV\right)=E_{total}\left(system\right)-\sum_{m}n_{m}E_{i}\left(m\right),$$

where $E_{total}$ is the total energy of the nanodiscs under consideration, $n_{m}$ is the number of specific atoms in the nanodisc, and $E_{i}\left(m\right)$ is the total energy of the m-th isolated atom, with *m* standing for Hydrogen (H), Carbon (C), Silicon (Si), Boron (B), or Nitrogen (N).

**Figure 7:**
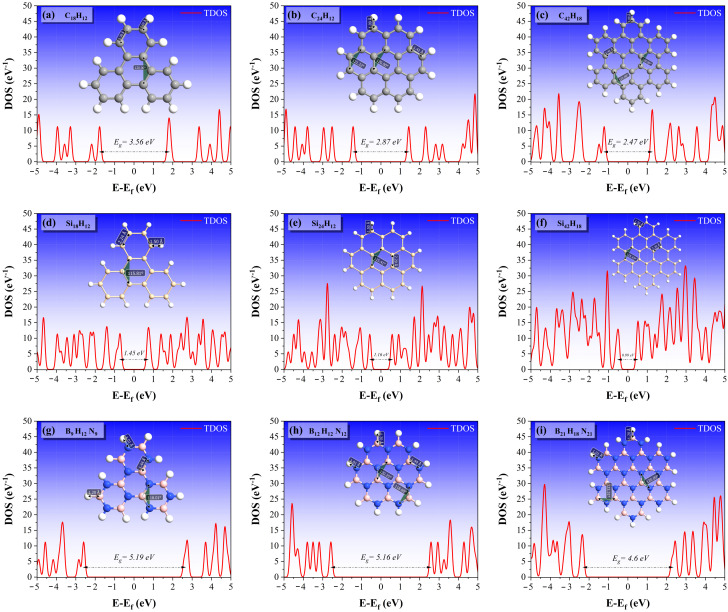
Total density of states (TDOS) of representative AT, ZH, and AH nanodiscs for (**a**–**c**) graphene, (**d**–**f**) silicene, and (**g**–**i**) h-BN. The optimized structures are shown inside the plots.

First, we calculate the electronic properties of armchair trigonal (AT), zigzag hexagonal (ZH), and armchair hexagonal (AH) nanodiscs. Figure 7 illustrates the corresponding total density of states (TDOS). Structure of the nanodiscs after full relaxation is also shown in the plots. It is evident from Figure 7 that there are no zero-energy states in these nanodiscs, and a rather wide band-gap appears in all the structures. According to Figure 7a–c, AT, ZH, and AH nanodiscs of graphene have a gap of 3.56 eV, 2.87 eV, and 2.47 eV, respectively. From Figure 7, it also follows that the band-gap decreases with an increasing number of atoms in the structures. The silicene nanodiscs display semiconductor behavior, with a small band-gap of 1.45 eV, 1.16 eV, and 0.99 eV for the AT, ZH, and AH nanodiscs. These gaps are smaller than those in the corresponding graphene nanodiscs. Finally, Figure 7g–i indicate very wide band-gaps of 5.19 eV, 5.16 eV, and 4.6 eV for the h-BN nanodiscs, which prove the insulating behavior in all cases.

**Figure 8:**
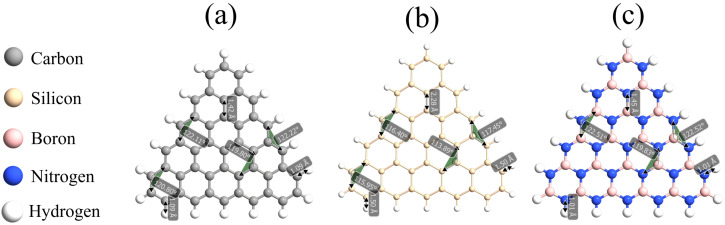
The optimized and minimized structure of the (**a**) graphene, (**b**) silicene, and (**c**) h-BN zigzag trigonal nanodiscs with a width of N = 4.

Another type of nanodisc we study is the zigzag trigonal nanodisc. As already mentioned above, they are constructed by connecting several hexagons. The shape of these nanodiscs is shown in Figure 2a. In turn, in Figure 8 we show the relaxed structures of zigzag trigonal nanodiscs of graphene (a), silicene (b), and h-BN (c) for a width corresponding to N = 4. Note that each zigzag trigonal nanodisc can be simply classified by the number N, which in turn determines the total number of atoms in the structure following the relation

(3) $$N_{x}=N^{2}+9N+12,$$

where $N_{x}$ is the total number of atoms in each nanodisc, including the numbers of Carbon (*C*), Silicon (Si), Boron (*B*), Nitrogen (*N*), and Hydrogen (*H*) atoms.

**Figure 9:**
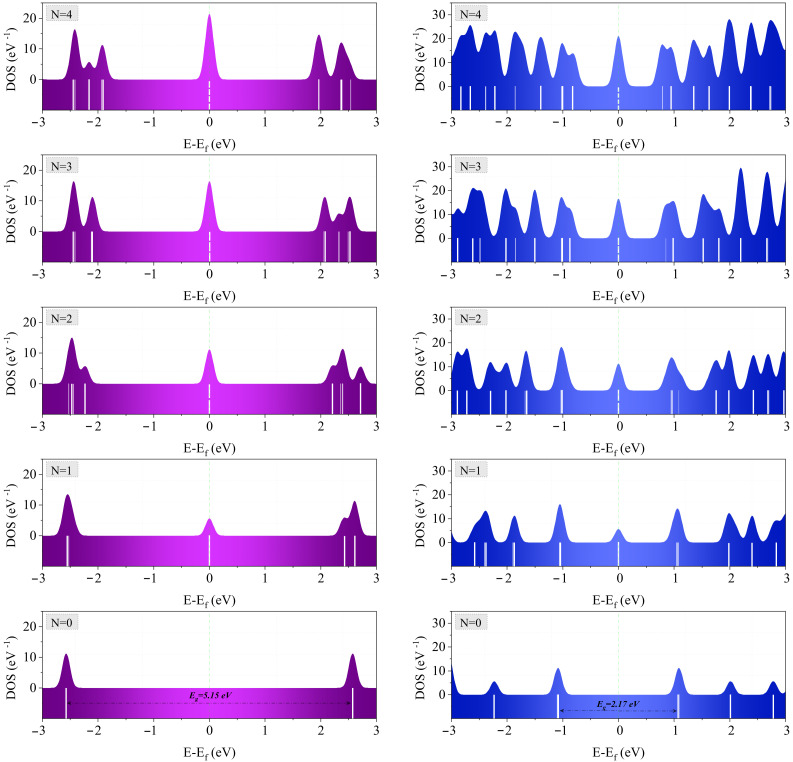
Total density of states (TDOS) and energy spectrum of the (**left**) graphene, and (**right**) silicene zigzag trigonal nanodiscs for different widths from N = 0 to N = 4. The dashed lines on colored bars indicate the degeneracy of energy levels.

From the results obtained for various types and shapes of nanodiscs, it is evident that the zero-energy states emerge rather rarely. Among different nanodiscs, the zigzag trigonal ones with N ≥1 have zero-energy states. However, the zero-energy states do not appear in the h-BN zigzag trigonal nanodiscs, independently of the type of edges (armchair or zigzag ones). Hence, h-BN displays insulator behavior in all cases of nanodiscs. We have also found a similar absence of zero-energy states in the armchair trigonal nanodiscs, see Figure 7. Figure 9 shows the total density of states as well as the full energy spectrum for the graphene and silicene zigzag trigonal nanodiscs. The half-filled zero-energy states occur only for N ≥1. Furthermore, another crucial point is that the degeneracy in the zigzag trigonal nanodiscs can be predicted and designed. This degeneracy originates from the overlap of the HOMO and LUMO, and increases proportionally with the width of nanodiscs (N). Furthermore, we note that the zero-energy states are well separated from other states even for large values of N. Therefore, one can describe the low-energy physics near the Fermi energy ϵ=0 by taking into account only the zero-energy states.

## 4. Transport Properties

**Figure 10:**
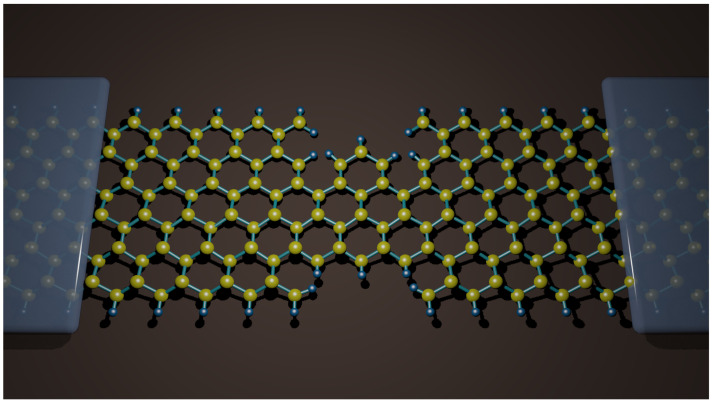
Schematic of the device with two semi-infinite electrodes and the zigzag trigonal nanodisc (N = 2) as the central part, connected to the screening region of the graphene and silicene nanoribbons. Yellow balls represent C or Si atoms for graphene or silicene case, respectively, whereas blue balls represent H atoms.

To gain further insight into the peculiar electronic properties of zigzag trigonal nanodiscs, we determine their transport properties. To do this, we consider a two-probe molecular junction consisting of two semi-infinite nanoribbons of graphene or silicene as electrodes, which are connected to a zigzag trigonal nanodisc as the central region, as shown schematically in Figure 10.

**Figure 11:**
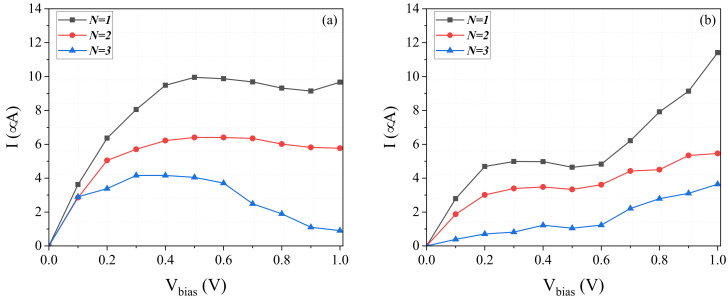
The current–voltage (I−V) characteristics for (**a**) graphene-based and (**b**) silicene-based junctions.

Current–voltage, I−V, characteristics of the junction with graphene and silicene zigzag trigonal nanodiscs of different widths are shown in Figure 11. In the small bias region, the current grows roughly linearly with the voltage, indicating a non-zero density of states around the Fermi level (zero energy). This behavior holds in both graphene and silicene junctions with N =1,2,3, and indicates the presence of zero-energy states in the corresponding graphene and silicene zigzag trigonal nanodiscs. From Figure 11a for the graphene nanodevice, one can clearly see that above a certain voltage the increase in current with voltage slows down and then, at higher voltages, it remains around 10 μA and 6 μA for the widths of N =1 and N =2, respectively. In other words, the corresponding differential conductance is close to zero. However, for the device with a width of N =3, the current drops to the level of 1 mA, revealing a relatively large negative differential conductance. It is also noteworthy that the magnitude of current decreases with increasing width N of the nanodiscs.

Figure 11b shows the I−V curves of the silicene nanodevices. At small voltages, the behavior of these curves is similar to that in graphene junctions. However, at higher voltages the current grows monotonously with voltage (contrary to graphene junctions), roughly up to 12 μA, 6 μA, and 4 μA for widths of N =1 to N =3, respectively. Similarly to graphene, the current decreases with increasing width, N, of the zigzag trigonal nanodiscs in the central region.

**Figure 12:**
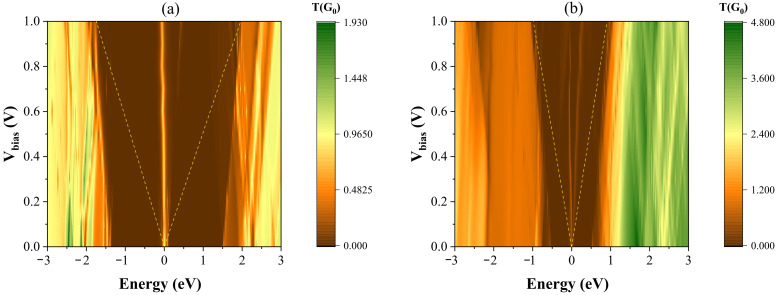
The total transmission spectrum as a function of the applied bias voltage and electron energy for the devices containing (**a**) graphene, (**b**) silicene zigzag trigonal nanodiscs (N = 2) as a central region and sandwiched between two semi-infinite electrodes. The dashed yellow lines represent the bias window.

In order to gain an intuitive representation of quantum transport in the devices under consideration, we plot in Figure 12 the maps of the transmission spectrum (T(E)) for the zigzag trigonal nanodiscs of width N = 2 (as representative) as a function of the applied bias voltage and electron energy. Magnitude of the transmission is displayed as a color scale (the right columns), and the Fermi level is set to be zero in both figures. The yellow dashed lines show the bias window, in which the transmission T(E) contributes to the current in the device. From these figures, behavior of the current–voltage characteristics can be clearly accounted for by the changes in T(E) under the bias voltage. As anticipated from the electronic properties, the transmission spectrum shows the metallic behavior near the Fermi energy due to the existence of the zero-energy states.

## 5. Conclusions

We have used density functional theory to study electronic properties of finite nanoribbons and of various nanodiscs based on 2D materials such as graphene, silicene, and hexagonal boron nitride. The main objective of this analysis was a search for zero-energy states. Such states play an important role as their presence is connected with certain topological aspects of a given structure. In addition, such states also determine metallic behavior of the nanosystem. The zero-energy states exist rather rarely, and we have found them, e.g., in trigonal nanodiscs of graphene and silicene with zigzag edges. In turn, there are no zero-energy states in boron nitride nanodiscs and nanoribbons. For this material, all nanodiscs are in the insulator state, with a relatively large energy gap at the Fermi level.

The conclusions based on the density functional theory have also been confirmed by calculating transport properties (IV characteristics) of the corresponding junctions based on graphene, silicene, and boron nitride. These junctions include the corresponding nanoribbons as external electrodes that are connected to a specific nanodisc. The latter plays the role of the central (scattering) region.

## Figures and Tables

**Table 1:** The binding energy (in units of eV) for different selected 2D materials.

Nanodiscs	Graphene	Silicene	h-BN
Zigzag Trigonal N = 0	−74.690	−46.341	−70.727
Zigzag Trigonal N = 1	−147.23	−87.756	−142.230
Zigzag Trigonal N = 2	−237.902	−138.524	−231.058
Zigzag Trigonal N = 3	−346.729	−198.634	−337.234
Zigzag Trigonal N = 4	−473.695	−268.040	−460.753
Armchair Trigonal	−203.079	−120.520	−193.244
Zigzag Hexagonal	−257.614	−148.402	−245.214
Armchair Hexagonal	−439.875	−250.579	−419.759

## Data Availability

The data supporting reported results are available from the corresponding author, A.D., upon reasonable request.

## Supplementary Materials

The following supporting information can be downloaded at: https://www.mdpi.com/article/10.3390/molecules27072228/s1. The Supplementary Material containing optimized structure of the Nanodiscs (Section I) and comment on choosen mesh cutoff (Section II) and many-body dispersion corrections (Section III) .
