# Supplementary material for: First Principle Study on Electronic and Transport Properties of Finite-Length Nanoribbons and Nanodiscs for Selected Two-Dimensional Materials
Source: Molecules. 2022 Mar 29;27(7):2228. doi: 10.3390/molecules27072228 (PMC9000399; doi:10.3390/molecules27072228)
Supplement: Supplementary file 1 [file molecules-27-02228-s001.zip › molecules-1639385-supplementary.pdf]

SUPPLEMENTARY MATERIAL

First principle study on electronic and transport properties of finite length nanoribbons and nanodisks for selected two-dimensional materials

Mirali Jafari,[1] and Anna Dyrdał[1*]

[1]*Department of Mesoscopic Physics, ISQI, Faculty of Physics, Adam Mickiewicz University in Poznań,*
ul. Uniwersytetu Poznanskiego 2, 61-614 Poznan, Poland

* Correspondence: adyrdal@amu.edu.pl

Table of Contents

I. The optimized structure of the Nanodiscs 1
II. Comment on the mesh cutoff chosen in calculations 19
III. Comment on many-body dispersion correction 19

I. The optimized structure of the Nanodiscs

1. Graphene Nanodiscs

We have used the simple cubic lattice type with a=25 A. The positions of atoms are defined in the Cartesian coordinates.

1.1 Benzene

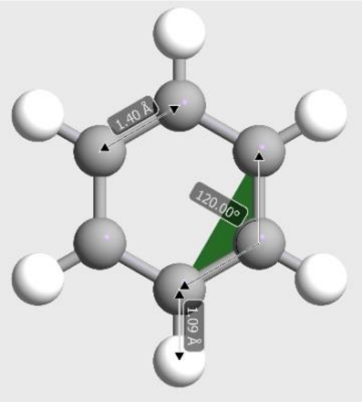

Element	x (Å)	y (Å)	z (Å)
Carbon	11.2902	13.1985	12.5
Carbon	12.5	11.103	12.5
Carbon	13.7098	13.1985	12.5
Carbon	11.2902	11.8015	12.5
Carbon	12.5	13.897	12.5
Carbon	13.7098	11.8015	12.5
Hydrogen	10.348	13.7425	12.5
Hydrogen	12.5	10.0151	12.5
Hydrogen	14.652	13.7425	12.5
Hydrogen	10.348	11.2575	12.5
Hydrogen	12.5	14.9849	12.5
Hydrogen	14.652	11.2575	12.5

1.2 Zigzag hexagonal nanodisc

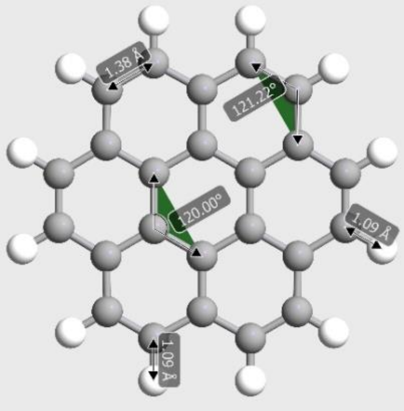

Element	x (Å)	y (Å)	z (Å)	Element	x (Å)	y (Å)	z (Å)	Element	x (Å)	y (Å)	z (Å)
Carbon	8.81678	13.1878	12.5	Carbon	8.81678	11.8122	12.5	Hydrogen	7.8746	13.7349	12.5
Carbon	10.0627	15.3459	12.5	Carbon	10.0324	13.9247	12.5	Hydrogen	9.1178	15.8883	12.5
Carbon	10.0324	11.0753	12.5	Carbon	11.2541	16.0337	12.5	Hydrogen	11.2568	7.87681	12.5
Carbon	11.2654	13.2128	12.5	Carbon	10.0627	9.65415	12.5	Hydrogen	15.8822	15.8883	12.5
Carbon	12.5	15.3493	12.5	Carbon	11.2654	11.7872	12.5	Hydrogen	13.7432	7.87681	12.5
Carbon	11.2541	8.96628	12.5	Carbon	12.5	13.9256	12.5	Hydrogen	17.1254	13.7349	12.5
Carbon	12.5	11.0744	12.5	Carbon	13.7459	16.0337	12.5	Hydrogen	7.8746	11.2651	12.5
Carbon	13.7346	13.2128	12.5	Carbon	12.5	9.65069	12.5	Hydrogen	11.2568	17.1232	12.5
Carbon	14.9373	15.3459	12.5	Carbon	13.7346	11.7872	12.5	Hydrogen	9.1178	9.11175	12.5
Carbon	13.7459	8.96628	12.5	Carbon	14.9676	13.9247	12.5	Hydrogen	13.7432	17.1232	12.5
Carbon	14.9676	11.0753	12.5	Carbon	14.9373	9.65415	12.5	Hydrogen	15.8822	9.11175	12.5
Carbon	16.1832	13.1878	12.5	Carbon	16.1832	11.8122	12.5	Hydrogen	17.1254	11.2651	12.5

1.3 Armchair trigonal nanodisc

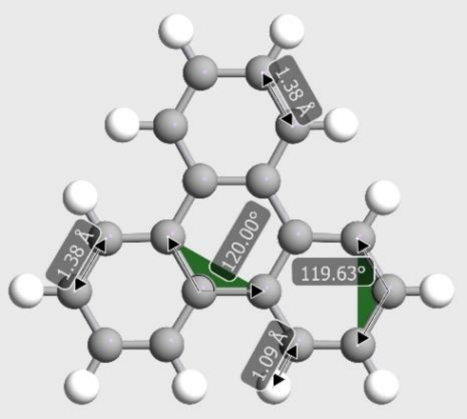

Element	x (Å)	y (Å)	z (Å)	Element	x (Å)	y (Å)	z (Å)
Carbon	12.5	15.3546	9.42632	Carbon	12.5	11.7885	13.139
Carbon	12.5	11.0297	9.43532	Carbon	12.5	13.8851	14.3812
Carbon	12.5	13.2314	10.6399	Carbon	12.5	11.7991	15.5845

Carbon	12.5	15.3555	11.8346	Hydrogen	12.5	15.8892	8.47881
Carbon	12.5	8.94455	10.6402	Hydrogen	12.5	11.55	8.48212
Carbon	12.5	11.0571	11.8722	Hydrogen	12.5	15.9209	12.7617
Carbon	12.5	13.2115	13.139	Hydrogen	12.5	7.8567	10.6511
Carbon	12.5	11.1149	14.3812	Hydrogen	12.5	10.0291	14.4073
Carbon	12.5	13.2009	15.5845	Hydrogen	12.5	13.7542	16.5212
Carbon	12.5	13.9703	9.43532	Hydrogen	12.5	13.45	8.48212
Carbon	12.5	16.0554	10.6402	Hydrogen	12.5	17.1433	10.6511
Carbon	12.5	9.64543	9.42632	Hydrogen	12.5	9.11084	8.47881
Carbon	12.5	11.7686	10.6399	Hydrogen	12.5	9.07906	12.7617
Carbon	12.5	13.9429	11.8722	Hydrogen	12.5	14.9709	14.4073
Carbon	12.5	9.64448	11.8346	Hydrogen	12.5	11.2458	16.5212

1.4 Armchair hexagonal nanodisc

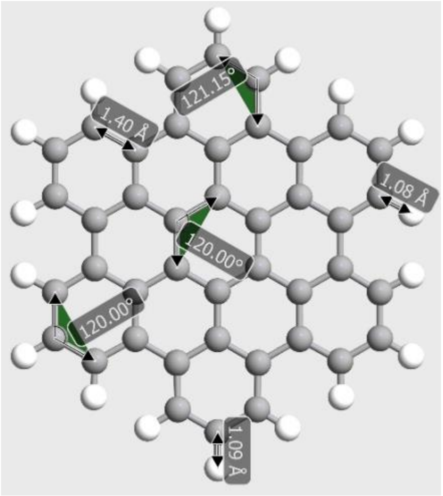

Element	x (Å)	y (Å)	z (Å)	Element	x (Å)	y (Å)	z (Å)	Element	x (Å)	y (Å)	z (Å)	Element	x (Å)	y (Å)	z (Å)
Carbon	7.58022	15.3404	12.5	Carbon	13.7299	8.91541	12.5	Carbon	12.5	13.923	12.5	Hydrogen	8.75099	7.88126	12.5
Carbon	7.58022	11.0471	12.5	Carbon	14.9813	11.0674	12.5	Carbon	13.7299	16.0846	12.5	Hydrogen	14.6254	18.0561	12.5
Carbon	8.78073	13.2272	12.5	Carbon	16.2193	13.2272	12.5	Carbon	11.2984	7.51286	12.5	Hydrogen	12.5	5.73145	12.5
Carbon	10.0106	15.3574	12.5	Carbon	17.4198	15.3404	12.5	Carbon	12.5	9.63479	12.5	Hydrogen	18.3617	15.8843	12.5
Carbon	11.2984	17.4871	12.5	Carbon	16.2181	8.96578	12.5	Carbon	13.7323	11.7885	12.5	Hydrogen	16.249	7.88126	12.5
Carbon	8.78185	8.96578	12.5	Carbon	17.4198	11.0471	12.5	Carbon	14.9813	13.9326	12.5	Hydrogen	18.3744	11.5627	12.5
Carbon	10.0187	11.0674	12.5	Carbon	7.58022	13.9529	12.5	Carbon	16.2181	16.0342	12.5	Hydrogen	6.62558	13.4373	12.5
Carbon	11.2677	13.2115	12.5	Carbon	8.78185	16.0342	12.5	Carbon	13.7016	7.51286	12.5	Hydrogen	8.75099	17.1187	12.5
Carbon	12.5	15.3652	12.5	Carbon	7.58022	9.65955	12.5	Carbon	14.9894	9.64257	12.5	Hydrogen	6.6383	9.11573	12.5
Carbon	13.7016	17.4871	12.5	Carbon	8.78073	11.7728	12.5	Carbon	16.2193	11.7728	12.5	Hydrogen	12.5	19.2685	12.5
Carbon	11.2701	8.91541	12.5	Carbon	10.0187	13.9326	12.5	Carbon	17.4198	13.9529	12.5	Hydrogen	10.3746	6.94388	12.5
Carbon	12.5	11.077	12.5	Carbon	11.2701	16.0846	12.5	Carbon	17.4198	9.65955	12.5	Hydrogen	16.249	17.1187	12.5
Carbon	13.7323	13.2115	12.5	Carbon	12.5	18.1809	12.5	Hydrogen	6.6383	15.8843	12.5	Hydrogen	14.6254	6.94388	12.5
Carbon	14.9894	15.3574	12.5	Carbon	10.0106	9.64257	12.5	Hydrogen	6.62558	11.5627	12.5	Hydrogen	18.3744	13.4373	12.5
Carbon	12.5	6.81909	12.5	Carbon	11.2677	11.7885	12.5	Hydrogen	10.3746	18.0561	12.5	Hydrogen	18.3617	9.11573	12.5

1.5 Zigzag trigonal nanodisc (N=4)

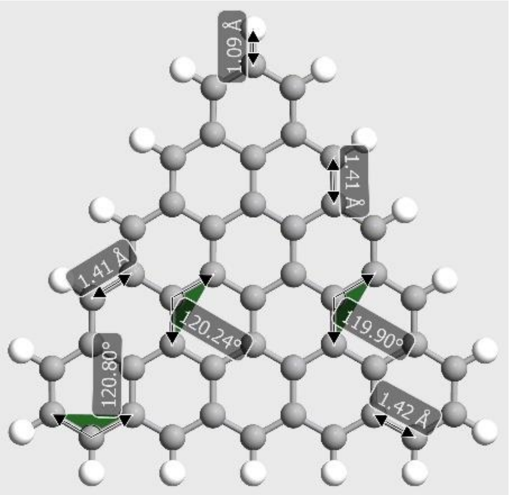

Element	x (Å)	y (Å)	z (Å)	Element	x (Å)	y (Å)	z (Å)	Element	x (Å)	y (Å)	z (Å)	Element	x (Å)	y (Å)	z (Å)
Carbon	6.34837	8.93044	12.5	Carbon	12.5	6.82803	12.5	Carbon	10.0342	9.6595	12.5	Hydrogen	7.87057	13.7559	12.5
Carbon	7.58374	11.0735	12.5	Carbon	13.7325	8.95026	12.5	Carbon	11.2656	11.7958	12.5	Hydrogen	9.10636	15.8906	12.5
Carbon	8.81497	13.2107	12.5	Carbon	14.9633	11.0822	12.5	Carbon	12.5	13.9304	12.5	Hydrogen	10.3457	18.0306	12.5
Carbon	10.0502	15.3456	12.5	Carbon	16.185	13.2107	12.5	Carbon	13.742	16.0683	12.5	Hydrogen	7.56048	5.74378	12.5
Carbon	11.2884	17.487	12.5	Carbon	14.9665	6.83032	12.5	Carbon	11.2674	7.5097	12.5	Hydrogen	14.6543	18.0306	12.5
Carbon	7.55991	6.83199	12.5	Carbon	16.1978	8.94819	12.5	Carbon	12.5	9.6578	12.5	Hydrogen	10.0334	5.74043	12.5
Carbon	8.80219	8.94819	12.5	Carbon	17.4163	11.0735	12.5	Carbon	13.7344	11.7958	12.5	Hydrogen	15.8936	15.8906	12.5
Carbon	10.0367	11.0822	12.5	Carbon	17.4401	6.83199	12.5	Carbon	14.9784	13.9373	12.5	Hydrogen	12.5	5.73754	12.5
Carbon	11.2691	13.2169	12.5	Carbon	18.6516	8.93044	12.5	Carbon	13.7326	7.5097	12.5	Hydrogen	17.1294	13.7559	12.5
Carbon	12.5	15.353	12.5	Carbon	6.35883	7.53751	12.5	Carbon	14.9658	9.6595	12.5	Hydrogen	14.9666	5.74044	12.5
Carbon	13.7116	17.487	12.5	Carbon	7.56173	9.66617	12.5	Carbon	16.211	11.8024	12.5	Hydrogen	18.3602	11.6184	12.5
Carbon	10.0336	6.83032	12.5	Carbon	8.78904	11.8024	12.5	Carbon	16.1963	7.51494	12.5	Hydrogen	17.4395	5.74379	12.5
Carbon	11.2676	8.95026	12.5	Carbon	10.0216	13.9373	12.5	Carbon	17.4383	9.66617	12.5	Hydrogen	19.5938	9.47505	12.5
Carbon	12.5	11.0831	12.5	Carbon	11.258	16.0683	12.5	Carbon	18.6412	7.53751	12.5	Hydrogen	5.41652	6.99343	12.5
Carbon	13.7309	13.2169	12.5	Carbon	12.5	18.1744	12.5	Hydrogen	5.40623	9.47505	12.5	Hydrogen	12.5	19.2625	12.5
Carbon	14.9498	15.3456	12.5	Carbon	8.80374	7.51494	12.5	Hydrogen	6.6398	11.6184	12.5	Hydrogen	19.5835	6.99343	12.5

2. Silicene Nanodiscs

We have used the simple cubic lattice (a=25 Angstrom or larger as noticed). The positions of atoms are given in the Cartesian coordinates.

2.1 Zigzag trigonal nanodisc N=0:

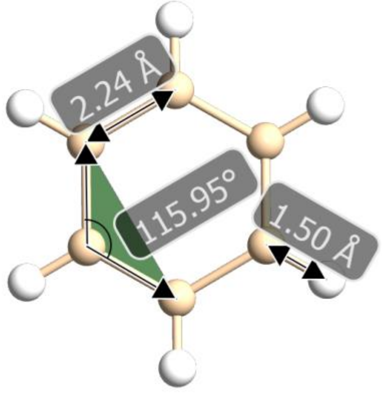

Element	x (Å)	y (Å)	z (Å)
Silicon	10.6106	11.3869	12.7313
Silicon	12.4811	14.695	12.7289
Silicon	14.4105	11.4187	12.7254
Silicon	10.5902	13.5803	12.2728
Silicon	12.5185	10.3059	12.2687
Silicon	14.3914	13.6143	12.2745
Hydrogen	9.339	10.6383	12.4638
Hydrogen	12.4679	16.1688	12.4524
Hydrogen	15.6933	10.6936	12.4462
Hydrogen	9.30667	14.3061	12.5462
Hydrogen	12.532	8.83117	12.5387
Hydrogen	15.6611	14.3617	12.5564

2.2 Zigzag trigonal nanodisc N=1:

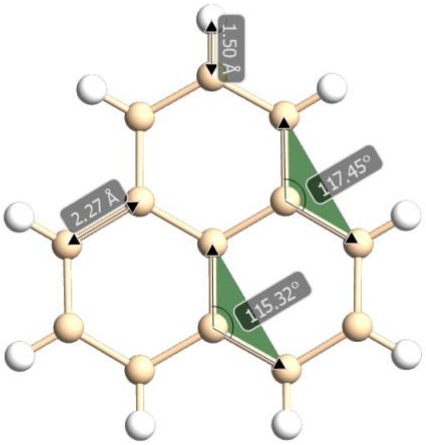

**Element**	**x (Å)**	**y (Å)**	**z (Å)**	**Element**	**x (Å)**	**y (Å)**	**z (Å)**
Silicon	8.69298	9.72621	12.8107	Silicon	14.4504	8.63271	12.2834
Silicon	10.5667	13.0504	12.719	Silicon	16.3448	11.946	12.2985
Silicon	12.4744	16.3512	12.8044	Hydrogen	7.4029	8.97475	12.6677
Silicon	12.5075	9.72341	12.715	Hydrogen	12.4677	17.8446	12.6504
Silicon	14.4174	13.0668	12.7234	Hydrogen	17.6155	9.01928	12.6404
Silicon	16.3194	9.75999	12.7986	Hydrogen	7.38449	12.6379	12.6632
Silicon	8.64762	11.9117	12.3012	Hydrogen	9.30259	15.9995	12.645
Silicon	10.5717	15.2796	12.2908	Hydrogen	10.5841	7.15536	12.6513
Silicon	10.5726	8.61376	12.294	Hydrogen	15.6487	16.0312	12.6649
Silicon	12.4975	11.9477	12.1893	Hydrogen	14.4525	7.17298	12.6344
Silicon	14.3898	15.2973	12.3029	Hydrogen	17.6033	12.6806	12.6597

2.3 Zigzag trigonal nanodisc N=2: (Bulk size = 30 A)

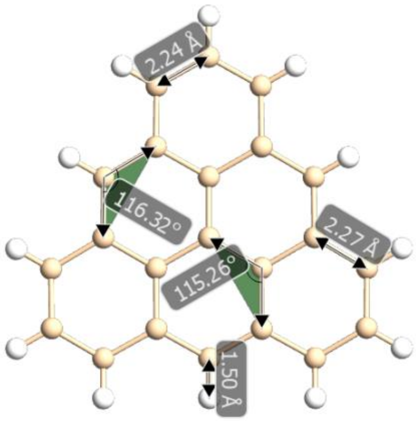

Element	x (Å)	y (Å)	z (Å)	Element	x (Å)	y (Å)	z (Å)
Silicon	9.24632	10.5523	15.2909	Silicon	15.0025	9.45405	14.7091
Silicon	11.135	13.8858	15.2214	Silicon	16.9295	12.7698	14.7165
Silicon	13.0544	17.2196	15.2187	Silicon	18.828	16.104	14.7213
Silicon	14.9873	20.5276	15.2872	Silicon	18.8752	9.45863	14.7818
Silicon	13.0774	10.5335	15.2175	Silicon	20.7756	12.7595	14.7892
Silicon	14.9933	13.8817	15.1954	Hydrogen	7.95773	9.80417	15.1128
Silicon	16.9255	17.2256	15.2237	Hydrogen	14.982	22.0174	15.1003
Silicon	16.9232	10.5387	15.2178	Hydrogen	22.0469	9.82988	15.0956
Silicon	18.8557	13.8953	15.2222	Hydrogen	7.95315	13.466	15.1288
Silicon	20.7576	10.5712	15.2835	Hydrogen	9.88626	16.8201	15.0509
Silicon	9.22201	12.7383	14.7969	Hydrogen	11.816	20.1825	15.1035
Silicon	11.1566	16.0927	14.7152	Hydrogen	11.1308	7.98256	15.1195
Silicon	13.0867	19.4495	14.7851	Hydrogen	18.1621	20.1914	15.1175
Silicon	11.1304	9.44456	14.7915	Hydrogen	15.0035	7.98919	15.0415
Silicon	13.062	12.7638	14.7183	Hydrogen	20.0935	16.8385	15.0599
Silicon	14.9921	16.1139	14.7163	Hydrogen	18.8832	7.99348	15.102
Silicon	16.894	19.4564	14.7918	Hydrogen	22.0427	13.4943	15.1153

2.4 Zigzag trigonal nanodisc N=3: (Bulk size = 35 A)

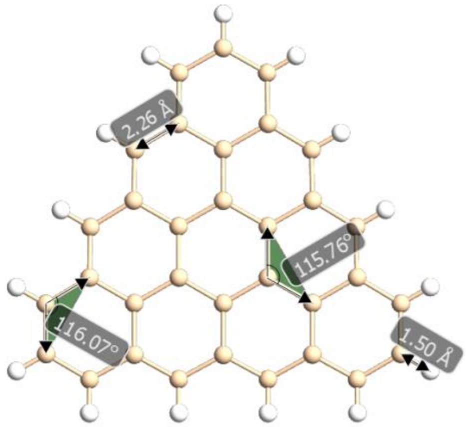

Element	x (Å)	y (Å)	z (Å)	Element	x (Å)	y (Å)	z (Å)
Silicon	9.79105	13.5693	17.2807	Silicon	15.57	14.7118	17.7679
Silicon	11.7286	16.9102	17.2376	Silicon	17.4983	18.0569	17.7676
Silicon	13.6645	20.2722	17.231	Silicon	19.4384	21.411	17.7656
Silicon	15.5912	23.6266	17.2737	Silicon	17.5021	11.3393	17.7364
Silicon	11.6973	10.2714	17.2809	Silicon	19.4319	14.7105	17.769
Silicon	13.6354	13.5908	17.3057	Silicon	21.3823	18.0704	17.7417
Silicon	15.5621	16.9443	17.2963	Silicon	21.3653	11.3559	17.7607
Silicon	17.4968	20.2925	17.3068	Silicon	23.309	14.7206	17.7641
Silicon	19.3987	23.628	17.2766	Silicon	25.1975	11.3887	17.7479
Silicon	15.563	10.2834	17.2343	Hydrogen	8.51421	14.2902	17.6012
Silicon	17.5006	13.5894	17.2987	Hydrogen	10.4539	17.6556	17.5093
Silicon	19.4346	16.9435	17.299	Hydrogen	12.3841	21.0074	17.5027
Silicon	21.3259	20.278	17.2385	Hydrogen	14.3344	24.3787	17.5912
Silicon	19.4418	10.2844	17.2338	Hydrogen	11.6799	8.8051	17.5979
Silicon	21.3697	13.597	17.3055	Hydrogen	20.6634	24.3694	17.5968
Silicon	23.2683	16.9218	17.2377	Hydrogen	15.5704	8.80755	17.5056
Silicon	23.3074	10.2827	17.2724	Hydrogen	22.6048	21.0158	17.5102
Silicon	25.2097	13.5782	17.2729	Hydrogen	19.4403	8.80758	17.5019
Silicon	9.80879	11.38	17.7542	Hydrogen	24.5451	17.6634	17.5103
Silicon	11.694	14.7099	17.7635	Hydrogen	23.3266	8.81715	17.5889
Silicon	13.6112	18.0651	17.7354	Hydrogen	26.4858	14.2989	17.5954
Silicon	15.5513	21.4063	17.7611	Hydrogen	8.52252	10.6405	17.5339
Silicon	17.4953	24.7112	17.7487	Hydrogen	17.4999	26.1949	17.5295
Silicon	13.6382	11.3498	17.7626	Hydrogen	26.484	10.6488	17.5292

2.5 Zigzag trigonal nanodisc N=4: (Bulk size = 40 A)

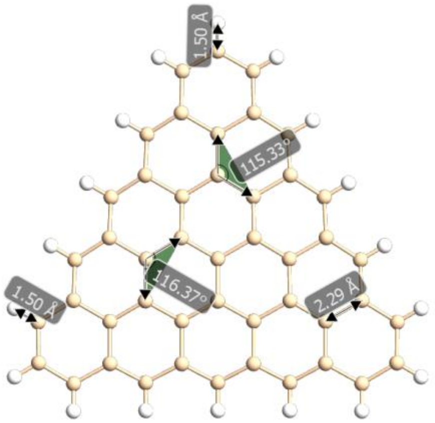

Element	x (Å)	y (Å)	z (Å)	Element	x (Å)	y (Å)	z (Å)
Silicon	10.365	14.3967	19.7319	Silicon	16.1225	15.5308	20.4045
Silicon	12.3445	17.7166	19.5481	Silicon	18.0484	18.8961	20.5167
Silicon	14.2794	21.068	19.4823	Silicon	19.9968	22.2469	20.4041
Silicon	16.2092	24.4215	19.5466	Silicon	21.9419	25.5763	20.1968
Silicon	18.0927	27.7964	19.7244	Silicon	18.0747	12.1673	20.1385
Silicon	12.2691	11.1043	19.7281	Silicon	20.0002	15.519	20.5177
Silicon	14.2178	14.4286	19.7961	Silicon	21.9502	18.8996	20.5147
Silicon	16.1601	17.7616	19.921	Silicon	23.8888	22.2417	20.1392
Silicon	18.0845	21.0986	19.9195	Silicon	21.9242	12.1708	20.1398
Silicon	19.9946	24.4477	19.7972	Silicon	23.878	15.5374	20.4043
Silicon	21.8928	27.7981	19.7308	Silicon	25.8179	18.9072	20.1357
Silicon	16.1332	11.1645	19.5467	Silicon	25.7847	12.1853	20.1918
Silicon	18.0744	14.4502	19.9206	Silicon	27.7362	15.5563	20.1924
Silicon	20.0003	17.7712	20.1359	Silicon	29.6084	12.215	20.2379
Silicon	21.9115	21.1021	19.9202	Hydrogen	9.0945	15.1235	20.0575
Silicon	23.7811	24.4257	19.5529	Hydrogen	11.0532	18.4757	19.6641
Silicon	20.0009	11.1702	19.4843	Hydrogen	12.9838	21.818	19.6152
Silicon	21.9281	14.4533	19.9223	Hydrogen	14.9078	25.1625	19.6628
Silicon	23.8402	17.7673	19.9191	Hydrogen	16.8264	28.5338	20.0429
Silicon	25.717	21.0744	19.4832	Hydrogen	12.2646	9.64039	20.0517
Silicon	23.8678	11.173	19.5456	Hydrogen	23.1577	28.5364	20.052
Silicon	25.7835	14.4365	19.7957	Hydrogen	16.1459	9.66687	19.6621
Silicon	27.6565	17.7233	19.545	Hydrogen	25.0847	25.1627	19.6691
Silicon	27.7287	11.1077	19.7236	Hydrogen	20.0065	9.6728	19.6162
Silicon	29.6337	14.3973	19.7283	Hydrogen	27.0131	21.8232	19.6182
Silicon	10.3923	12.2152	20.2432	Hydrogen	23.8594	9.67457	19.655
Silicon	12.2659	15.5498	20.1953	Hydrogen	28.9478	18.4807	19.6661
Silicon	14.1817	18.901	20.1383	Hydrogen	27.7272	9.64387	20.0475
Silicon	16.1057	22.2375	20.1379	Hydrogen	30.9055	15.1231	20.0525

Silicon	18.0455	25.5747	20.194	Hydrogen	9.10108	11.4707	20.0762
Silicon	19.9916	28.8695	20.2368	Hydrogen	19.9947	30.3596	20.0648
Silicon	14.2162	12.1772	20.1937	Hydrogen	30.8996	11.471	20.069

2.6 Armchair trigonal nanodisc (bulk size: 30 A)

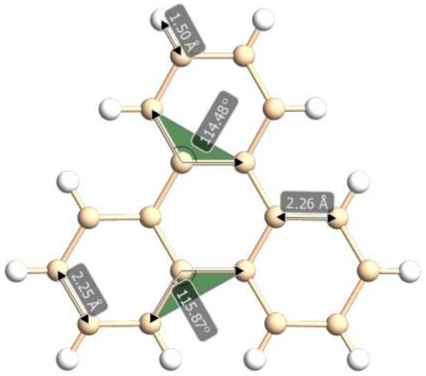

Element	x (Å)	y (Å)	z (Å)	Element	x (Å)	y (Å)	z (Å)
Silicon	17.2403	10.2156	15.3147	Silicon	16.1153	15.9741	14.7649
Silicon	20.5372	12.1016	15.1668	Silicon	12.816	17.8783	14.6853
Silicon	10.5739	10.1941	15.1762	Silicon	16.098	19.7905	14.8331
Silicon	13.8768	12.099	15.2358	Hydrogen	16.4958	8.93199	15.0932
Silicon	17.2407	14.0249	15.2349	Hydrogen	22.005	12.0689	14.8603
Silicon	10.5746	13.9957	15.3102	Hydrogen	9.81449	8.93586	14.8721
Silicon	13.8918	15.9708	15.2359	Hydrogen	9.82925	15.2772	15.0786
Silicon	17.1771	17.8904	15.3141	Hydrogen	18.6594	17.9054	15.0818
Silicon	13.8766	19.7854	15.1775	Hydrogen	13.1647	21.0693	14.8715
Silicon	19.4222	10.1804	14.8223	Hydrogen	20.1781	8.9219	15.1286
Silicon	12.757	10.2156	14.6896	Hydrogen	13.4939	8.92931	14.9213
Silicon	16.1262	12.1009	14.7639	Hydrogen	20.1855	15.2624	14.9181
Silicon	19.4312	13.9863	14.6858	Hydrogen	7.995	12.0874	15.1278
Silicon	9.46436	12.1159	14.8237	Hydrogen	11.3321	17.8754	14.9069
Silicon	12.7656	14.0237	14.7642	Hydrogen	16.8035	21.0781	15.1396

2.7 Armchair hexagonal nanodisc (Box size: 40A)

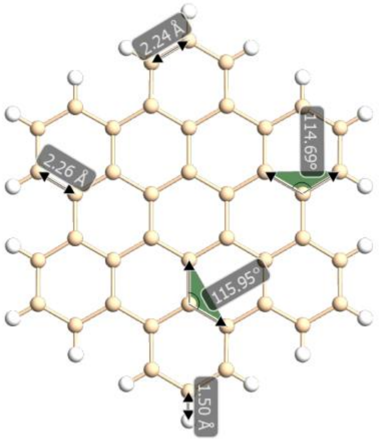

Element	x (Å)	y (Å)	z (Å)	Element	x (Å)	y (Å)	z (Å)
Silicon	12.2946	24.4363	19.8091	Silicon	20.0013	22.225	20.2518
Silicon	12.3197	17.7441	19.739	Silicon	21.9247	25.5868	20.2495
Silicon	14.1994	21.1175	19.75	Silicon	18.1015	12.2192	20.2642
Silicon	16.1253	24.4561	19.7501	Silicon	20.0021	15.5339	20.218
Silicon	18.0958	27.7808	19.7369	Silicon	21.9309	18.89	20.2516
Silicon	14.2154	14.4653	19.7358	Silicon	23.8712	22.2392	20.2173
Silicon	16.138	17.7674	19.7805	Silicon	25.7913	25.539	20.2639
Silicon	18.0767	21.1111	19.7477	Silicon	21.8918	12.2184	20.2607
Silicon	19.9967	24.4683	19.7828	Silicon	23.8757	15.5355	20.2506
Silicon	21.8876	27.7836	19.7398	Silicon	25.8028	18.8798	20.2503
Silicon	18.0741	14.4152	19.7505	Silicon	27.6878	22.2531	20.2617
Silicon	20.0054	17.777	19.7481	Silicon	27.7028	15.5551	20.1891
Silicon	21.9293	21.115	19.7491	Hydrogen	11.0188	25.1729	20.0888
Silicon	23.8727	24.4689	19.7503	Hydrogen	11.0391	18.4895	19.9811
Silicon	19.9957	11.1071	19.8095	Hydrogen	16.8102	28.5193	19.9764
Silicon	21.9294	14.4153	19.7507	Hydrogen	14.2297	12.9832	19.9766
Silicon	23.8723	17.7653	19.7832	Hydrogen	23.1716	28.5236	19.9806
Silicon	25.8024	21.1253	19.7503	Hydrogen	19.9974	9.63384	20.0886
Silicon	27.6999	24.4504	19.8122	Hydrogen	28.9774	25.1831	20.0938
Silicon	25.7945	14.4661	19.7367	Hydrogen	25.7943	12.9834	19.9761
Silicon	27.6883	17.7524	19.7395	Hydrogen	28.9729	18.492	19.979
Silicon	12.3118	22.2397	20.2641	Hydrogen	11.0314	21.4923	20.0249
Silicon	14.2049	25.5227	20.261	Hydrogen	14.2085	27.0046	20.0206
Silicon	12.3039	15.5471	20.1932	Hydrogen	11.0259	14.8126	19.9167
Silicon	14.2042	18.873	20.2503	Hydrogen	19.9899	30.3662	19.9115
Silicon	16.1319	22.2281	20.2184	Hydrogen	16.8139	11.4842	20.0249
Silicon	18.0667	25.5844	20.2499	Hydrogen	25.7922	27.0217	20.0244
Silicon	19.9905	28.8929	20.1905	Hydrogen	23.1757	11.4787	20.0198

Silicon	16.1311	15.5396	20.2484	Hydrogen	28.9733	21.5151	20.0218
Silicon	18.0799	18.8882	20.2527	Hydrogen	28.9812	14.824	19.9072

2.8 Zigzag hexagonal nanodisc (Bulk size: 30 A)

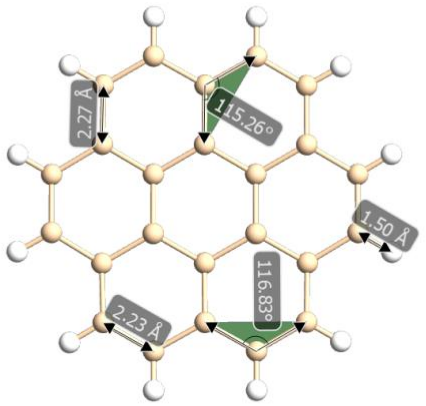

Element	x (Å)	y (Å)	z (Å)	Element	x (Å)	y (Å)	z (Å)
Silicon	6.74042	13.5993	12.2748	Silicon	14.4576	18.0376	12.7238
Silicon	8.69039	16.9573	12.2673	Silicon	12.5177	8.04007	12.7321
Silicon	8.63115	10.2601	12.275	Silicon	14.4348	11.377	12.7508
Silicon	10.573	13.6064	12.2391	Silicon	16.3534	14.7185	12.7515
Silicon	12.5174	16.9567	12.2563	Silicon	16.3487	8.05542	12.7181
Silicon	10.5776	6.95302	12.2749	Silicon	18.28	11.4173	12.7239
Silicon	12.5	10.2686	12.2391	Hydrogen	5.46258	14.342	12.5326
Silicon	14.4276	13.6067	12.2492	Hydrogen	7.41206	17.6966	12.5426
Silicon	16.3448	16.9377	12.2885	Hydrogen	10.5818	5.47496	12.5323
Silicon	14.4607	6.96286	12.2673	Hydrogen	17.6337	17.658	12.5451
Silicon	16.3737	10.2775	12.2562	Hydrogen	14.462	5.48616	12.5427
Silicon	18.271	13.6015	12.2884	Hydrogen	19.5393	14.3578	12.5454
Silicon	6.73077	11.4189	12.7397	Hydrogen	5.44301	10.6961	12.4666
Silicon	8.65191	14.736	12.7322	Hydrogen	10.5866	19.525	12.4651
Silicon	10.5805	18.0463	12.7181	Hydrogen	7.41449	7.28131	12.4665
Silicon	8.68431	8.03491	12.7395	Hydrogen	14.456	19.5131	12.4557
Silicon	10.5653	11.3768	12.7609	Hydrogen	17.6323	7.32121	12.4651
Silicon	12.5002	14.7278	12.751	Hydrogen	19.557	10.6781	12.4557

3. H-BN Nanodiscs

We have used the simple cubic lattice (a=25 Angstrom or larger as noticed). The positions of atoms are given in the Cartesian coordinates.

3.1 Zigzag trigonal nanodisc (N=0)

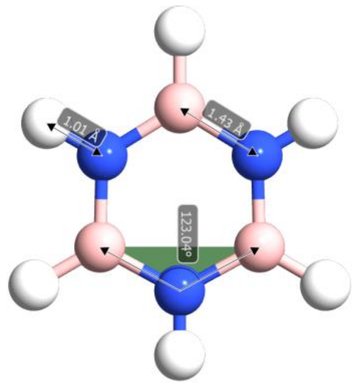

Element	x (Å)	y (Å)	z (Å)
Boron	11.2409	11.6565	12.5
Boron	12.5	13.8373	12.5
Boron	13.7591	11.6565	12.5
Nitrogen	11.2789	13.0885	12.5
Nitrogen	12.5	10.9735	12.5
Nitrogen	13.7211	13.0885	12.5
Hydrogen	10.2016	11.0565	12.5
Hydrogen	12.5	15.0374	12.5
Hydrogen	14.7984	11.0565	12.5
Hydrogen	10.4034	13.5939	12.5
Hydrogen	12.5	9.96256	12.5
Hydrogen	14.5966	13.5939	12.5

3.2 Zigzag trigonal nanodisc (N=1)

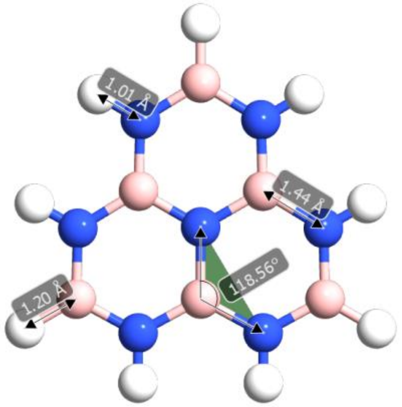

Element	x (Å)	y (Å)	z (Å)
Boron	9.98041	10.5658	12.5
Boron	11.2431	12.7462	12.5
Boron	12.5	14.9299	12.5
Boron	12.5	10.5692	12.5
Boron	13.7569	12.7462	12.5
Boron	15.0196	10.5658	12.5
Nitrogen	10.014	11.9948	12.5
Nitrogen	11.2793	14.1865	12.5
Nitrogen	11.2346	9.8805	12.5

Nitrogen	12.5	12.0206	12.5
Nitrogen	13.7207	14.1865	12.5
Nitrogen	13.7654	9.8805	12.5
Nitrogen	14.986	11.9948	12.5
Hydrogen	8.94029	9.96541	12.5
Hydrogen	12.5	16.131	12.5
Hydrogen	16.0597	9.96541	12.5
Hydrogen	9.13519	12.4956	12.5
Hydrogen	10.4061	14.697	12.5
Hydrogen	11.229	8.869	12.5
Hydrogen	14.5939	14.697	12.5
Hydrogen	13.771	8.869	12.5
Hydrogen	15.8648	12.4956	12.5

3.3 Zigzag trigonal nanodisc (N=2)

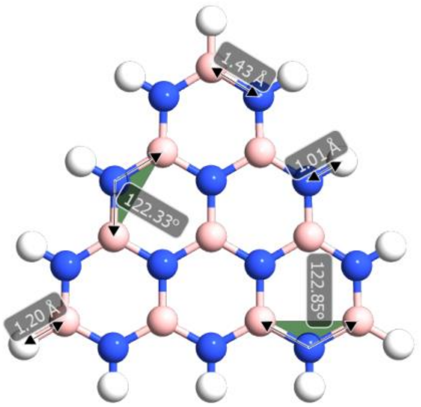

Element	x (Å)	y (Å)	z (Å)	Element	x (Å)	y (Å)	z (Å)
Boron	8.72172	9.47693	12.5	Nitrogen	12.5	8.78966	12.5
Boron	9.98637	11.6573	12.5	Nitrogen	13.7553	10.9337	12.5
Boron	11.2441	13.8357	12.5	Nitrogen	14.9844	13.0929	12.5
Boron	12.5	16.0212	12.5	Nitrogen	15.0248	8.79307	12.5
Boron	11.2423	9.48198	12.5	Nitrogen	16.2438	10.9046	12.5
Boron	12.5	11.6583	12.5	Hydrogen	7.68066	8.87604	12.5
Boron	13.7559	13.8357	12.5	Hydrogen	12.5	17.2234	12.5
Boron	13.7577	9.48198	12.5	Hydrogen	17.3193	8.87604	12.5
Boron	15.0136	11.6573	12.5	Hydrogen	7.87734	11.4053	12.5
Boron	16.2783	9.47693	12.5	Hydrogen	9.13821	13.5993	12.5
Nitrogen	8.75618	10.9046	12.5	Hydrogen	10.4078	15.7883	12.5
Nitrogen	10.0156	13.0929	12.5	Hydrogen	9.96947	7.78164	12.5
Nitrogen	11.2809	15.2778	12.5	Hydrogen	14.5922	15.7883	12.5
Nitrogen	9.97523	8.79307	12.5	Hydrogen	12.5	7.77658	12.5

Nitrogen	11.2447	10.9337	12.5	Hydrogen	15.8618	13.5993	12.5
Nitrogen	12.5	13.108	12.5	Hydrogen	15.0305	7.78164	12.5
Nitrogen	13.7191	15.2778	12.5	Hydrogen	17.1227	11.4053	12.5

3.4 Zigzag trigonal nanodisc (N=3)

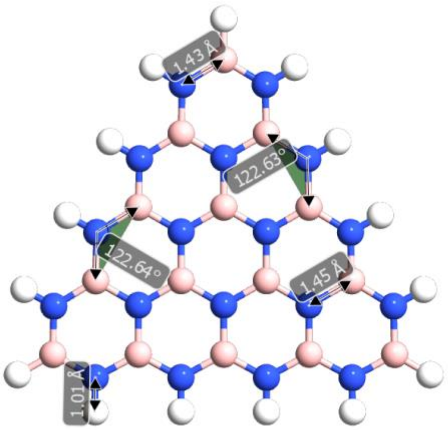

Element	x (Å)	y (Å)	z (Å)	Element	x (Å)	y (Å)	z (Å)
Boron	7.45898	8.38133	12.5	Nitrogen	11.2401	7.69932	12.5
Boron	8.72515	10.5623	12.5	Nitrogen	12.5	9.84334	12.5
Boron	9.98619	12.7432	12.5	Nitrogen	13.7546	12.0164	12.5
Boron	11.2443	14.9258	12.5	Nitrogen	14.9813	14.1795	12.5
Boron	12.5	17.1129	12.5	Nitrogen	13.7599	7.69932	12.5
Boron	9.98087	8.38745	12.5	Nitrogen	15.0156	9.83959	12.5
Boron	11.2427	10.566	12.5	Nitrogen	16.2412	11.9972	12.5
Boron	12.5	12.7436	12.5	Nitrogen	16.2874	7.69519	12.5
Boron	13.7557	14.9258	12.5	Nitrogen	17.5085	9.81029	12.5
Boron	12.5	8.3891	12.5	Hydrogen	6.41656	7.77975	12.5
Boron	13.7573	10.566	12.5	Hydrogen	12.5	18.3167	12.5
Boron	15.0138	12.7432	12.5	Hydrogen	18.5834	7.77975	12.5
Boron	15.0191	8.38745	12.5	Hydrogen	6.61051	10.3081	12.5
Boron	16.2749	10.5623	12.5	Hydrogen	7.8823	12.5022	12.5
Boron	17.541	8.38133	12.5	Hydrogen	9.14303	14.6859	12.5
Nitrogen	7.49148	9.81029	12.5	Hydrogen	10.407	16.8842	12.5
Nitrogen	8.75881	11.9972	12.5	Hydrogen	8.70341	6.68331	12.5
Nitrogen	10.0187	14.1795	12.5	Hydrogen	14.593	16.8842	12.5
Nitrogen	11.2789	16.3705	12.5	Hydrogen	11.2393	6.68773	12.5
Nitrogen	8.71259	7.69519	12.5	Hydrogen	15.857	14.6859	12.5
Nitrogen	9.98441	9.83959	12.5	Hydrogen	13.7607	6.68773	12.5
Nitrogen	11.2454	12.0164	12.5	Hydrogen	17.1177	12.5022	12.5
Nitrogen	12.5	14.1969	12.5	Hydrogen	16.2966	6.68331	12.5
Nitrogen	13.7211	16.3705	12.5	Hydrogen	18.3895	10.3081	12.5

3.5 Zigzag trigonal nanodisc (N=4) (Bulk size: 30 A)

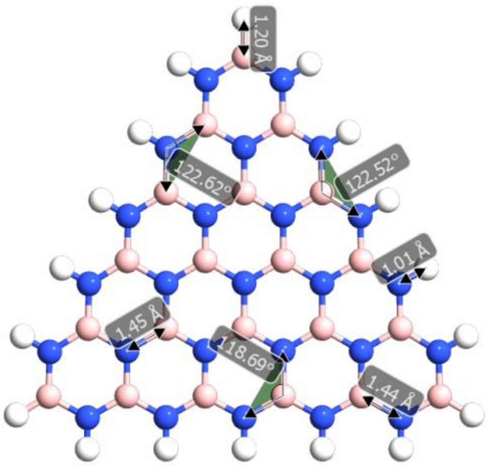

Element	x (Å)	y (Å)	z (Å)	Element	x (Å)	y (Å)	z (Å)
Boron	8.70028	9.79058	15	Nitrogen	12.4822	9.11057	15
Boron	9.96816	11.9713	15	Nitrogen	13.7432	11.2542	15
Boron	11.2302	14.1515	15	Nitrogen	15	13.428	15
Boron	12.4884	16.3308	15	Nitrogen	16.2541	15.6034	15
Boron	13.7454	18.5138	15	Nitrogen	17.48	17.7672	15
Boron	15	20.7023	15	Nitrogen	15	9.11113	15
Boron	11.2228	9.79829	15	Nitrogen	16.2568	11.2542	15
Boron	12.487	11.9769	15	Nitrogen	17.5109	13.4264	15
Boron	13.7442	14.1529	15	Nitrogen	18.7383	15.5864	15
Boron	15	16.3297	15	Nitrogen	17.5178	9.11057	15
Boron	16.2546	18.5138	15	Nitrogen	18.7722	11.25	15
Boron	13.7418	9.80112	15	Nitrogen	19.9977	13.4061	15
Boron	15	11.9777	15	Nitrogen	20.0452	9.10622	15
Boron	16.2558	14.1529	15	Nitrogen	21.2652	11.2194	15
Boron	17.5116	16.3308	15	Hydrogen	7.65832	9.18926	15
Boron	16.2582	9.80112	15	Hydrogen	15	21.9055	15
Boron	17.513	11.9769	15	Hydrogen	22.3417	9.18926	15
Boron	18.7698	14.1515	15	Hydrogen	7.85411	11.7175	15
Boron	18.7772	9.79829	15	Hydrogen	9.12587	13.9119	15
Boron	20.0318	11.9713	15	Hydrogen	10.3853	16.0922	15
Boron	21.2997	9.79058	15	Hydrogen	11.6437	18.2731	15
Nitrogen	8.73475	11.2194	15	Hydrogen	12.908	20.4714	15
Nitrogen	10.0023	13.4061	15	Hydrogen	9.94604	8.09447	15
Nitrogen	11.2617	15.5864	15	Hydrogen	17.092	20.4714	15
Nitrogen	12.52	17.7672	15	Hydrogen	12.4822	8.09869	15

Nitrogen	13.78	19.9583	15	Hydrogen	18.3563	18.2731	15
Nitrogen	9.95475	9.10622	15	Hydrogen	15	8.09921	15
Nitrogen	11.2278	11.25	15	Hydrogen	19.6147	16.0922	15
Nitrogen	12.4891	13.4264	15	Hydrogen	17.5178	8.09869	15
Nitrogen	13.7459	15.6034	15	Hydrogen	20.8741	13.9119	15
Nitrogen	15	17.7839	15	Hydrogen	20.054	8.09447	15
Nitrogen	16.22	19.9583	15	Hydrogen	22.1459	11.7175	15

3.6 Armchair trigonal nanodisc

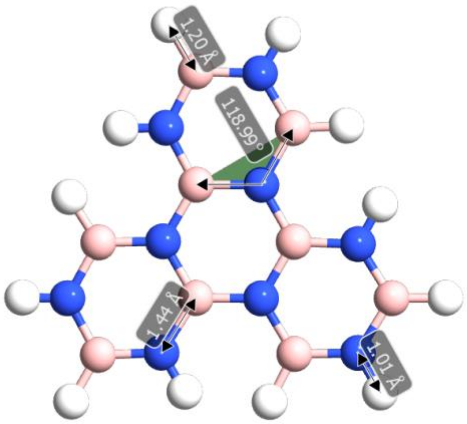

Element	x (Å)	y (Å)	z (Å)	Element	x (Å)	y (Å)	z (Å)
Boron	12.5	13.8534	9.36378	Nitrogen	12.5	13.1158	13.1387
Boron	12.5	16.0315	10.6087	Nitrogen	12.5	10.9826	14.4112
Boron	12.5	9.46016	9.3441	Nitrogen	12.5	13.0852	15.6256
Boron	12.5	11.6454	10.6133	Hydrogen	12.5	13.3004	8.29519
Boron	12.5	13.8397	11.8664	Hydrogen	12.5	17.2321	10.6128
Boron	12.5	9.47112	11.8529	Hydrogen	12.5	8.86356	8.30236
Boron	12.5	11.6573	13.1401	Hydrogen	12.5	8.82235	12.8661
Boron	12.5	13.8179	14.4033	Hydrogen	12.5	15.0199	14.4587
Boron	12.5	11.6506	15.6672	Hydrogen	12.5	11.0469	16.7048
Nitrogen	12.5	15.2783	9.3872	Hydrogen	12.5	15.7715	8.50446
Nitrogen	12.5	10.882	9.3935	Hydrogen	12.5	11.3981	8.52265
Nitrogen	12.5	13.1093	10.6041	Hydrogen	12.5	15.7738	12.6977
Nitrogen	12.5	15.2779	11.8152	Hydrogen	12.5	7.76794	10.6214
Nitrogen	12.5	8.77902	10.6072	Hydrogen	12.5	9.97039	14.3995
Nitrogen	12.5	10.9174	11.877	Hydrogen	12.5	13.603	16.4941

3.7 Armchair hexagonal nanodisc (Box size: 30A)

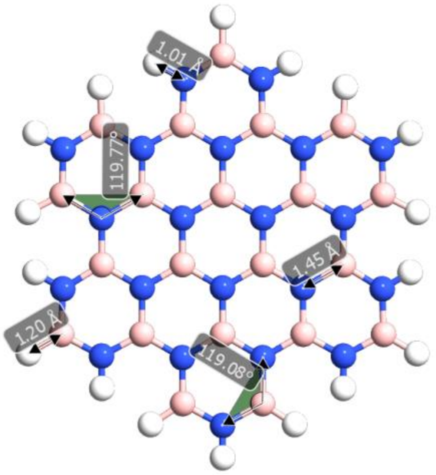

Element	x (Å)	y (Å)	z (Å)	Element	x (Å)	y (Å)	z (Å)
Boron	9.97686	16.3322	15	Nitrogen	16.2134	19.9494	15
Boron	11.2264	18.4967	15	Nitrogen	13.7404	11.2346	15
Boron	9.95778	11.9637	15	Nitrogen	15	13.4209	15
Boron	11.22	14.1411	15	Nitrogen	16.2593	15.6021	15
Boron	12.4814	16.3291	15	Nitrogen	17.5228	17.7862	15
Boron	13.7454	18.5154	15	Nitrogen	15	9.11234	15
Boron	15	20.6974	15	Nitrogen	16.2596	11.2346	15
Boron	12.4747	11.9681	15	Nitrogen	17.5165	13.4221	15
Boron	13.7406	14.1478	15	Nitrogen	18.7825	15.6044	15
Boron	15	16.3292	15	Nitrogen	19.9904	17.7564	15
Boron	16.2546	18.5154	15	Nitrogen	18.7876	11.287	15
Boron	13.7504	9.79596	15	Nitrogen	20.001	13.3887	15
Boron	15	11.9666	15	Hydrogen	8.90587	15.7878	15
Boron	16.2594	14.1478	15	Hydrogen	11.1625	19.6965	15
Boron	17.5186	16.3291	15	Hydrogen	8.91786	11.3635	15
Boron	18.7736	18.4967	15	Hydrogen	15	21.8982	15
Boron	16.2496	9.79596	15	Hydrogen	12.7433	9.14098	15
Boron	17.5253	11.9681	15	Hydrogen	18.8375	19.6965	15
Boron	18.78	14.1411	15	Hydrogen	17.2567	9.14098	15
Boron	20.0231	16.3322	15	Hydrogen	21.0941	15.7878	15
Boron	20.0422	11.9637	15	Hydrogen	21.0821	11.3635	15
Nitrogen	10.0096	17.7564	15	Hydrogen	9.13442	18.2616	15
Nitrogen	9.99896	13.3887	15	Hydrogen	9.1281	13.903	15
Nitrogen	11.2175	15.6044	15	Hydrogen	12.9054	20.4459	15
Nitrogen	12.4772	17.7862	15	Hydrogen	11.2226	10.2756	15
Nitrogen	13.7866	19.9494	15	Hydrogen	17.0946	20.4459	15
Nitrogen	11.2124	11.287	15	Hydrogen	15	8.10178	15

Nitrogen	12.4835	13.4221	15	Hydrogen	20.8656	18.2616	15
Nitrogen	13.7407	15.6021	15	Hydrogen	18.7774	10.2756	15
Nitrogen	15	17.7809	15	Hydrogen	20.8719	13.903	15

3.8 Zigzag hexagonal nanodisc

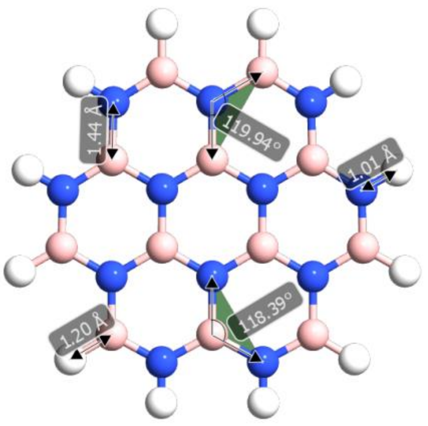

Element	x (Å)	y (Å)	z (Å)	Element	x (Å)	y (Å)	z (Å)
Boron	8.7284	11.6504	12.5	Nitrogen	12.5	10.9337	12.5
Boron	9.99114	13.8297	12.5	Nitrogen	13.7537	13.1053	12.5
Boron	11.247	16.013	12.5	Nitrogen	14.9686	15.2697	12.5
Boron	9.98134	9.48019	12.5	Nitrogen	13.7671	8.79929	12.5
Boron	11.2431	11.6555	12.5	Nitrogen	15.02	10.9264	12.5
Boron	12.5	13.8327	12.5	Nitrogen	16.2356	13.0751	12.5
Boron	13.753	16.013	12.5	Hydrogen	7.68416	11.0596	12.5
Boron	12.5	9.48418	12.5	Hydrogen	11.2368	17.2129	12.5
Boron	13.7569	11.6555	12.5	Hydrogen	8.94737	8.87159	12.5
Boron	15.0089	13.8297	12.5	Hydrogen	13.7632	17.2129	12.5
Boron	15.0187	9.48019	12.5	Hydrogen	16.0526	8.87159	12.5
Boron	16.2716	11.6504	12.5	Hydrogen	17.3158	11.0596	12.5
Nitrogen	8.76444	13.0751	12.5	Hydrogen	7.88286	13.5725	12.5
Nitrogen	10.0314	15.2697	12.5	Hydrogen	9.15971	15.7842	12.5
Nitrogen	9.98004	10.9264	12.5	Hydrogen	11.2231	7.78707	12.5
Nitrogen	11.2463	13.1053	12.5	Hydrogen	15.8403	15.7842	12.5
Nitrogen	12.5	15.2913	12.5	Hydrogen	13.7769	7.78707	12.5
Nitrogen	11.2329	8.79929	12.5	Hydrogen	17.1171	13.5725	12.5

II. Comment on the mesh cut-off chosen in calculations

The Quantum ATK is a numerical-orbital code, and it is easy to think that the real-space mesh determined by the mesh cut-off also controls the basis set functions of the atomic orbitals. This is one of the reasons why the values of the cut-off energy in Quantum ATK are bigger compared to plane-wave codes (e.g., Quantum Espresso).
Below, we present the convergence process of the mesh cut-off energy for Benzene (results for the other structures are in this range of energy). The converged value comes from the tolerance of total energy with the various mesh cut-off. The Figure below shows that the total energy is converged with the 10^-5 eV/ A accuracy at the 600 Ry.

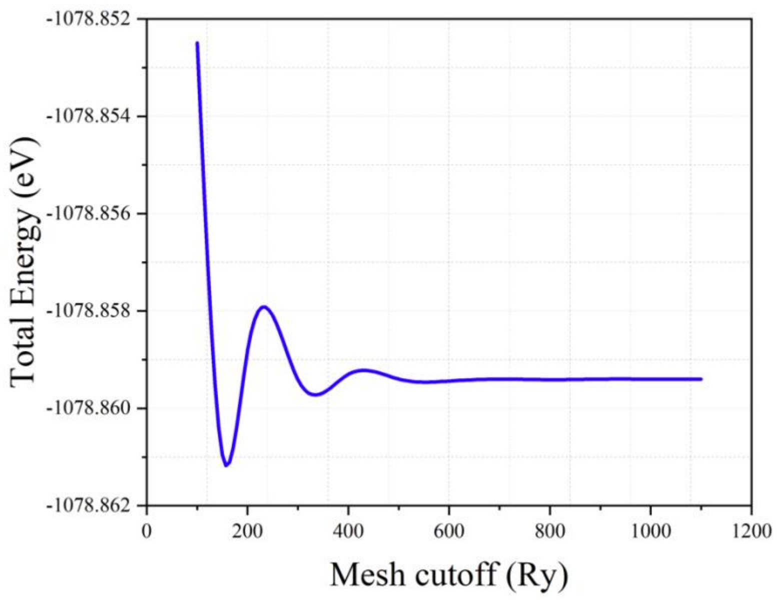

III. Comment on many-body dispersion correction

We have considered the many-body dispersion correction for most of the calculations (Van der Waals interaction within an appropriate semi-empirical modelling correction known as Grimme DFT-D3 approximation). There is no remarkable effect on the results near the Fermi energy for the systems under our considerations. For the DFT-D3 correction process, we have optimized the structure with the Grimme DFT-D3 with the Revised Perdew Burke Ernzerhof Exchange (RPBE) functional. Then, to obtain the DOS and Molecular energy spectrum, we have included the three-body term. In the Figure presented below, which is an example of the Benzene, one can clearly see that the difference in the total density of states of the system with and without dispersion correction is negligible, and one can neglect the dispersion correction in the calculations.

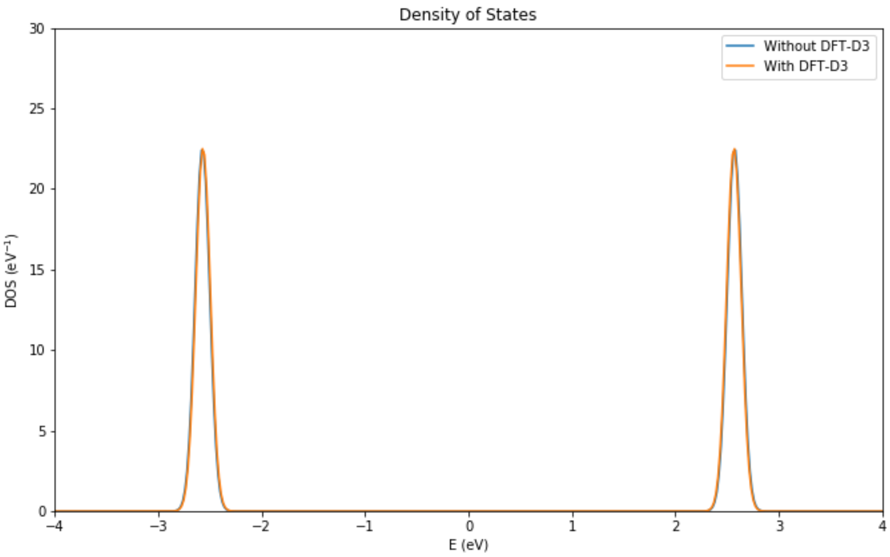
Density of States
Without DFT-D3
With DFT-D3
DOS (eV⁻¹)
E (eV)
30
25
20
15
10
5
0
−4
−3
−2
−1
0
1
2
3
4
